# Spinal Subdural Hematomas in a Normal Child without Trauma History: A Case Report

**Published:** 2019

**Authors:** Abdonaser FARZAN, Elham POURBAKHTYARAN, Toktam MOOSAVIAN, Hamidreza MOOSAVIAN

**Affiliations:** 1 Department of Neurosurgery, Mofid Hospital, Shahid Beheshti University of Medical Sciences, Tehran, Iran; 2 Mofid Children Hospital, Shahid Beheshti University of Medical Sciences, Tehran, Iran; 3 Department of Paediatric Neurology, Faculty of Medicine, Child Growth and Development Research Center, Isfahan University of Medical Sciences, Isfahan, Iran; 4 Department of Clinical Pathology, Faculty of Veterinary Medicine, University of Tehran, Tehran, Iran

**Keywords:** Acute spinal subdural hematoma, Acute paraplegia, Iran

## Abstract

Acute Spinal Subdural Hematoma (ASSH) is a rarely recognized condition that may result in severe irreversible neurologic complication. A 7-yr old girl presented to Neurology Department, Mofid Hospital, ShahidBeheshti University of Medical Sciences, Tehran, Iran with limping and pain in lower extremities and acute paraplegia without history of direct trauma. The patient had muscle weakness in lower limbs and was unable to bear weight. Deep Tendon Reflexes (DTR) in lower extremities had increased. Her MRI showed spinal subdural hematoma we reextended from T2 to T6. We performed laminectomy from T2 to T5 and about 70 cc of subdural hematoma was evacuated. One month after the surgery, the patient's neurological deficit resolved completely. The results showed the pivotal role of attention to clinical manifestation in acute spinal subdural hematoma and early diagnosis to prevent irreversible neurologic complication.

## Introduction

Acute spinal subdural hematoma (ASSH) is an uncommonly recognized condition that may result in severe irreversible neurologic complication. It usually presents with severe and localized back pain, rapidly progressive myelopathy and a poor outcome for functional recovery unless rapid surgical decompression is performed (1). 

Plain CT and MR can be used as complementary studies if needed to confirm the diagnosis of ASSH (2). Only a few cases were reported mostly due to trauma (3, 4) or accompanying diseases (5). A case of lumbar spinal subdural hematoma was reported in a patient with anticoagulant therapy (6). Another case was secondary to lumbar puncture during myelography (1). 

Here, we presente a 7-yr old girl with ASSH without obvious history of trauma.

## Case Presentation

A 7-yr old girl was transferred to Mofid Hospital, ShahidBeheshti University of Medical Sciences, Tehran, Iran with limping and pain in lower extremities and acute paraplegia. She had no history of direct trauma to back, no previous disease, no medication and no family history of neurologic disorders. 

**Figure 1:**
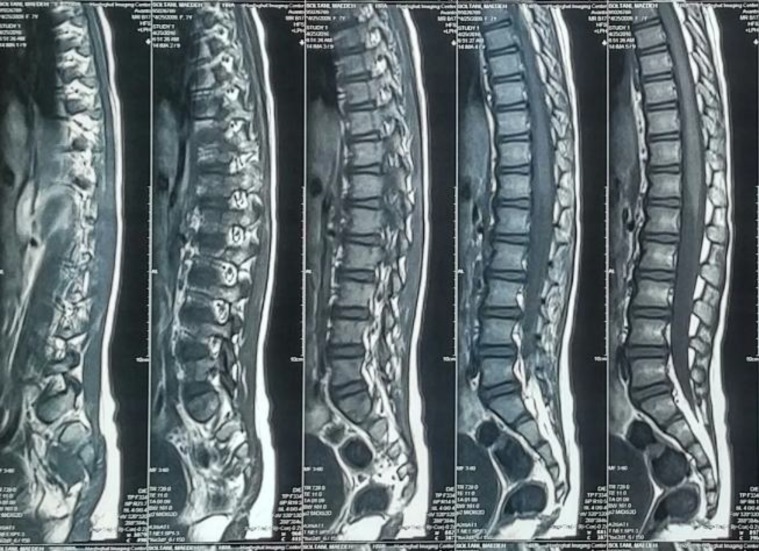
Sagital T1 weighted MRI showed spinal subdural hematoma extended from T2 to T6 (Arrow)

**Figure 2:**
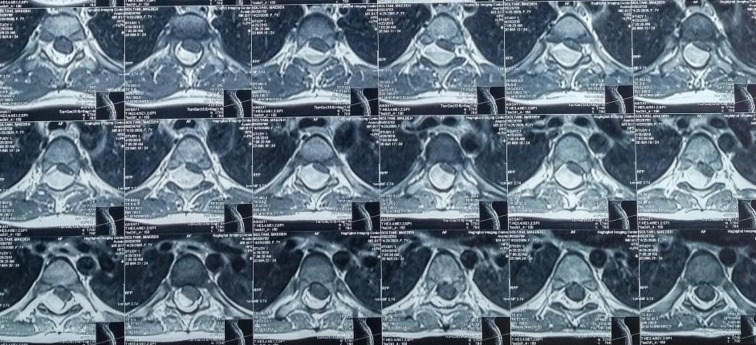
Axial T1 weighted MRI showed spinal subdural hematoma with compression effect to spinal cord (Arrow)

In her history, she had a ring dancing just for one time, and no history of trauma**.** In physical examination, she was good in general appearance with BP: 90/60, HR: 80, RR: 20, T: 36.5 axillary. She was unable to bear weight. Deep Tendon Reflexes (DTR) in lower extremities had increased with extensor plantar response. Cranial nerve and cerebellar examination were normal. Laboratory tests include complete blood count (CBC), prothrombin time, partial thromboplastin time, biochemistry and inflammatory markers were normal. Her MRI showed spinal subdural hematoma extended from T2 to T6 vertebra (Figures 1,2). The patient underwent laminectomy from T2 to T5 and about 70 cc of subdural hematoma was evacuated. One month after the surgery, the patient's neurological deficit resolved completely.

## Discussion

We presented a 7-yr old girl with ASSH and with lower extremities pain as well as weakness without history of trauma or medication. For diagnosis, in addition to history and physical examination, CT or MRI is needed. MRI and CT provide characteristic findings including: hyperdensity within the dural sac, clumping, location, and streaking of blood within the dural sac; an inhomogeneous and variable signal intensity on all MRI pulse sequences (2). 

In our patient, MRI showed subdural hematoma from T2 to T6 within the dural sac. Both conservatives and surgical management could be an option in management of ASSH considering the neurological course (5). In most of the patients, to prevent poor outcome, rapid surgical decompression should be performed (1). Only a few patients with ASSH were recovered spontaneously (3). Our patient underwent surgery and the neurologic deficits resolved completely after a month. 

**In conclusion, **results showed the importance of attention to clinical manifestation in ASSH and early diagnosis to prevent irreversible neurologic complication. MRI and plain CT are necessary and provide characteristic findings. 
